# The Aortic Watershed Phenomenon and Differential Hypoxemia During Peripheral Venoarterial ECMO: A Scoping Review of Clinical and Experimental Evidence

**DOI:** 10.31083/RCM52934

**Published:** 2026-09-30

**Authors:** Amin Hasheminia, Seif Kotb, Adham Elsherbini, Léonie Girard, Yasir Abu-Omar, Yakov Elgudin, Aron Frederik Popov, Mohammad El Diasty

**Affiliations:** ^1^Faculty of Medicine and Health Sciences, McGill University, Montreal, QC H3G 2M1, Canada; ^2^Case Western Reserve University, Cleveland, OH 44106, USA; ^3^Temerty Faculty of Medicine, University of Toronto, Toronto, ON M5S 1A8, Canada; ^4^Division of Cardiac Surgery, University Hospitals Cleveland Medical Center, Cleveland, OH 44106, USA; ^5^Department of Thoracic and Cardiovascular Surgery, Georg-August-University Goettingen, 37075 Goettingen, Germany

**Keywords:** venoarterial extracorporeal membrane oxygenation (VA-ECMO), watershed phenomenon, harlequin syndrome, computational fluid dynamics (CFD), cerebral oxygenation, differential hypoxemia

## Abstract

Extracorporeal membrane oxygenation (ECMO) is a widely used technology in cardiac surgery practice. A clinically significant yet underexplored presentation that occurs during peripheral femoro-femoral venoarterial ECMO (VA-ECMO) is differential hypoxemia (DH) or Harlequin syndrome. This phenomenon may occur when the aortic mixing interface shifts distally, and the coronary and supra-aortic circulations receive predominantly hypoxemic native blood. DH may reduce cerebral and myocardial oxygen delivery and produce clinically meaningful arterial partial pressure of carbon dioxide (PaCO_2_) gradients. This scoping review aimed to synthesize clinical and experimental evidence on the mechanisms, detection, management, and outcomes of DH during peripheral VA-ECMO. A scoping review of the watershed phenomenon during peripheral VA-ECMO was conducted using MEDLINE and EMBASE in accordance with the Preferred Reporting Items for Systematic Reviews and Meta-Analyses extension for Scoping Reviews (PRISMA-ScR) guidelines. The initial search identified 245 records. After screening, 16 studies were included. DH frequency ranged from 1.5% to 13.3%, but estimates varied substantially across case definitions, diagnostic thresholds, and cohort compositions. Regarding management, configuration-targeted strategies improved surrogates of upper-body oxygenation and perfusion, which included shifting venous drainage toward the superior vena cava/upper right atrium, in turn converting to veno-arteriovenous ECMO with internal jugular return (with flow-splitting control) and relocating arterial return to more proximal/antegrade sites. Detection extended beyond paired upper- and lower-body oximetry/arterial blood gases; reported approaches included contrast-enhanced ultrasound, Doppler mapping, and positional computed tomography angiography (CTA). Experimental models described why DH may persist despite higher ECMO flow as native output increases, and they highlight anatomy- and cannulation-dependent cerebral and coronary vulnerability. DH appears to be a predictable, physiology-driven complication best mitigated by modifying drainage position and/or circuit configuration rather than increasing ECMO flow. To further explore this phenomenon, adopting standardized definitions, investigating bedside localization tools, and conducting prospective comparative studies could be considered.

## 1. Introduction

Extracorporeal membrane oxygenation (ECMO) is a form of extracorporeal life support that can provide temporary cardiorespiratory support when conventional therapies are insufficient to maintain adequate systemic perfusion and gas exchange [1,2,3]. In adult practice, ECMO is commonly deployed in high-acuity settings, including intensive care units, cardiac surgical operating rooms, and other emergent procedural locations where rapid initiation of extracorporeal support may be required [2,4,5]. Contemporary indications include refractory cardiogenic shock and cardiac arrest requiring extracorporeal cardiopulmonary support, as well as selected patients with severe acute respiratory failure who remain hypoxemic despite optimized conventional management [3,4,5].

Venoarterial ECMO (VA-ECMO) provides hemodynamic support while simultaneously enabling oxygenation and carbon dioxide removal via a membrane oxygenator integrated into the extracorporeal circuit [1,4,5]. In emergency settings, the most common peripheral configuration uses femoral venous drainage and femoral arterial return, generating retrograde aortic flow from the iliac/femoral arterial system toward the aortic root [2,4,5]. Peripheral femoro-femoral VA-ECMO also predisposes to a configuration-specific complication characterized by competitive antegrade (native) and retrograde (ECMO) flows and the formation of a dynamic aortic mixing point, which is described in the literature using terms such as watershed, mixing interface/point, or “dual circulation” physiology [6,7,8]. This phenomenon arises when native left ventricular ejection meets retrograde oxygenated ECMO return, creating a variable anatomic mixing location that shifts with the balance among native cardiac output, ECMO flow, and systemic vascular conditions [4,6,7]. When pulmonary gas exchange remains poor while left-ventricular ejection persists or improves, the heart may eject relatively desaturated blood into the ascending aorta, and if the mixing point lies distal to the coronary ostia and/or supra-aortic branches, the myocardium and cerebral circulation may be preferentially supplied by hypoxemic native output, producing differential hypoxemia (DH) [4,6,7,8].

Importantly, the watershed phenomenon itself should be distinguished from clinically significant DH. The watershed phenomenon refers to the physiologic mixing interface created when retrograde oxygenated VA-ECMO return meets antegrade native left ventricular ejection [6,7]. This interface is expected to some degree in many peripheral femoro-femoral VA-ECMO configurations and is not necessarily pathologic. By contrast, DH occurs when pulmonary gas exchange remains impaired while native left ventricular ejection persists or recovers, allowing relatively desaturated native blood to supply the proximal aorta [6,7]. When the mixing interface lies distal to the coronary ostia and/or supra-aortic branches, the myocardium and brain may receive predominantly hypoxemic native blood despite apparently adequate distal oxygenation from femoral arterial ECMO return [6,7,8].

Clinically, DH is often recognized by a marked upper-body versus lower-body oxygenation gradient, commonly reflected in lower right radial/right upper-extremity (or right ear) saturation compared with lower-extremity values in femoral-return configurations [4,7,9]. This bedside pattern is also widely referred to as Harlequin syndrome or north-south syndrome, and it is clinically concerning because the same distribution that lowers right upper-extremity oxygenation may also indicate hypoxemic exposure of the brain and coronary circulation [6,7,9]. This distribution is clinically important because proximal aortic hypoxemia may expose both cerebral and myocardial territories to desaturated native blood even when lower-body oxygenation appears preserved [2,6]. Beyond oxygenation, dual-circulation physiology can produce clinically meaningful differences in carbon dioxide tension between vascular territories because carbon dioxide removal occurs across the membrane lung and may be nonuniform within the patient’s arterial circulation when mixing is incomplete [6,10]. Arterial partial pressure of carbon dioxide (PaCO_2_) gradients are specifically relevant to neurologic prognostication and to procedures such as brain-death apnea testing, where interpretation depends on confirming an adequate rise in blood carbon dioxide that reflects cerebral exposure [11,12,13].

Despite the impact of these implications, the literature on DH and watershed phenomenon is scattered across heterogeneous clinical reports and experimental approaches, including animal models, computational investigations, and benchtop flow studies, and often uses variable terminology, operational definitions, and monitoring endpoints [6,7,8]. The objective of this scoping review is therefore to consolidate and synthesize available clinical and experimental evidence on the VA-ECMO watershed phenomenon and DH, with an emphasis on mechanisms, detection/localization, management strategies, and reported outcomes, to support bedside decision-making and to identify priorities for future research.

## 2. Methods

### 2.1 Search Strategy

Reporting was structured using Preferred Reporting Items for Systematic Reviews and Meta-Analyses extension for Scoping Reviews (PRISMA-ScR) guidelines [14,15]. A systematic electronic search was conducted in MEDLINE and EMBASE from database inception to March 2025. The search combined terms related to watershed or mixing physiology with terms for extracorporeal support. The search included a combination of keywords, including “watershed”, “Harlequin”, “mixing cloud”, “north-south”, “mixing zone”, “ECMO”, “extracorporeal membrane oxygenation”, “ECLS”, and “extracorporeal life support”; Medical Subject Headings (MeSH) and corresponding database-specific subject headings were also applied to identify relevant literature. No publication-year restriction was applied. Complete database-specific search strategies are provided in **Supplementary Material**.

### 2.2 Inclusion and Exclusion Criteria

Eligible studies were: (1) primary research; (2) English-language; (3) clinical (cohorts, case series, case reports, feasibility studies) or experimental (computational modelling, mock circulation loop/*in vitro* systems) investigations; and (4) explicitly addressing a mixing/watershed phenomenon and/or DH (or differential gas exchange) in peripheral VA-ECMO. Because the same retrograde-perfusion “dual-circulation” physiology can be reproduced by closely related retrograde-perfusion mechanical circulatory support (MCS) configurations, studies that explicitly described a dynamic aortic mixing/watershed phenomenon relevant to VA-ECMO interpretation and decision-making were also included. Studies that did not meet these criteria, including non-primary literature and those not relevant to peripheral VA-ECMO mixing physiology or DH, were excluded.

### 2.3 Study Selection

Titles and abstracts were screened for relevance to peripheral VA-ECMO mixing/watershed physiology or clinically described DH/Harlequin physiology. Full texts were then assessed for eligibility by two independent reviewers (AH, SK). Any disagreements were resolved through discussion with a senior author (MD). Agreement statistics were not calculated. The selection process is presented in the PRISMA flow diagram in Fig. 1.

**Fig. 1.**
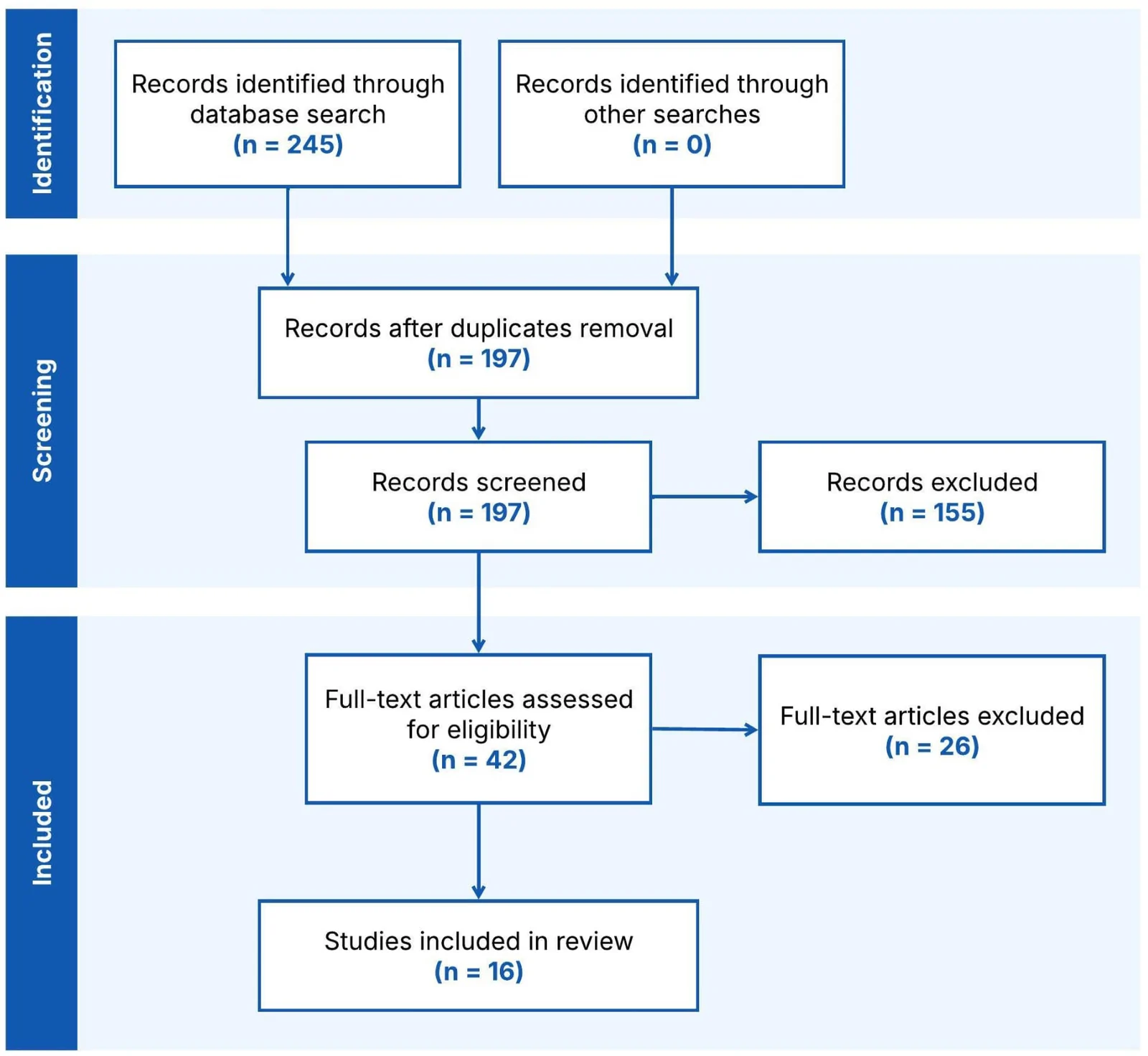
**Preferred Reporting Items for Systematic Reviews and Meta-Analyses (PRISMA) flow diagram**. Records identified through database searching (n = 245); records after duplicates removed (n = 197); records screened (n = 197); records excluded (n = 155); full-text articles assessed for eligibility (n = 42); full-text articles excluded (n = 26); studies included in review (n = 16).

### 2.4 Data Extraction and Synthesis

Data were extracted on study type, support configuration, description of the mixing/watershed phenomenon, terminology used for DH/Harlequin physiology, and key clinical or mechanistic findings. Included studies comprised 10 clinical investigations and 6 experimental investigations, reflecting two complementary emphases: (1) clinical recognition, detection/localization, management, and outcomes of DH/Harlequin physiology; and (2) mechanistic characterization of aortic mixing and the dependence of cerebral/coronary perfusion on native left ventricle (LV) output, cannulation geometry, and circuit flow distribution [16,17,18,19,20,21,22,23,24,25,26,27,28,29,30,31]. Clinical VA-ECMO studies and clinically adjacent MCS analogue reports were charted separately. Analogue evidence was used only to contextualize shared retrograde-flow physiology and imaging interpretation, rather than to estimate VA-ECMO incidence, outcomes, or treatment effects [19].

Table 1 (Ref. [16,17,18,19,20,21,22,24,26,28]) illustrates the general characteristics of these studies. Data were charted in a structured extraction sheet capturing study design, setting, population/model, cannulation configuration, and drainage/return sites; operational definitions for watershed/DH; detection/localization methods; interventions tested; and quantitative physiologic and clinical outcomes. Scoping review charting was used to support structured comparison across heterogeneous study types [14,15]. Evidence was synthesized descriptively and organized into clinical vs experimental domains. Interventions were grouped by mechanism (e.g., venous drainage repositioning, circuit reconfiguration to veno-arteriovenous (V-AV), flow-split control, arterial return relocation) to facilitate cross-study comparison of physiologic response signals and outcomes. No formal critical appraisal or risk-of-bias assessment was performed because the objective of this scoping review was to map heterogeneous clinical and experimental evidence rather than estimate pooled treatment effects.

**Table 1.** **Clinical studies included in the review**.

Study/First author	Design/setting	Sample size and population	Configuration	Key quantitative results and outcomes
*Du 2022 [19]	Case report; radiology (China)	2 adults on retrograde-perfusion MCS	LVAD LV→descending aorta; peripheral VA-ECMO + IABP	Dynamic aortic root/ascending filling defect on supine CTA resolved in right lateral decubitus; interpreted as watershed not thrombus; clinical stabilization reported
Contento 2020 [18]	Retrospective cohort; cardiac surgery/HTx unit (Italy)	60 VA-ECMO; Harlequin 8/60 (13.3%); Harlequin subgroup mean age 57 years, LVEF 29.8%, systolic PAP 81 mmHg	Femoro-femoral VA → V-AV with 15 Fr IJV return	Upper/lower PaO_2_ ratio 0.8 ± 0.1→0.95 ± 0.02; SaO_2_ ratio 0.83 ± 0.02→1.0; MAP ~50→~81 mmHg; lactate 5.3→~0.9 mmol/L; V-AV 5.3 ± 1.4 d; 7/8 VA→V-AV→VV then weaned after 13.5 ± 2.7 d VV; 1/8 returned to VA then weaned
Falk 2023 [20]	Retrospective cohort; ECMO center (Sweden)	472 VA-ECMO; fulminant DH 7/472 (1.5%); mean age 39.6 years; ARDS; SAVE score −3	Femoro-femoral VA with drainage moved IVC→SVC/upper RA	Upper-body SpO_2_ 54 ± 6.6→86 ± 6.6 (*p* < 0.001); pre-oxygenator sat 62 ± 8.9→74 ± 3.7 (*p* = 0.016); pre-pump pressure −66 ± 35→−31 ± 37 (*p* = 0.037); lactate 6.5→2.8 mmol/L at 24 h; 5/7 survived to discharge and 1-year; 2 died of intracerebral lesions
Buchtele 2020 [16]	Prospective feasibility/safety; 3 ICUs (Austria)	10 bifemoral VA-ECMO; median age 52; arrest 60%	Bifemoral VA; CEUS via drainage cannula	Watershed visualized in abdominal aorta 5/10; inferred proximal/root 5/10; no new neuro deficits/embolic CT findings reported; 5/10 weaned; 3/10 CPC 1–2
Cakici 2018 [17]	Retrospective V-AV series (Turkey)	12 V-AV; mean age 61.5 years; LVEF 33.3%	V-AV with IJV return and controllable split	Return split changed from ~70% IJV (2.59 ± 0.29 L/min) to 34.3 ± 7.4% (1.28 ± 0.36) with femoral arterial limb rising to 65.6 ± 7.4% (2.42 ± 0.39); PaO_2_ ratio 0.51 ± 0.07→0.93 ± 0.02; lactate 4.75→0.73 mmol/L; pH 7.11→7.39; 8/12 weaned; 4 deaths (sepsis/MOF)
Gangahanumaiah 2021 [22]	Retrospective case series (Australia)	3 SCI cases among 504 VA-ECMO runs (0.6%)	Femoro-femoral VA	Deficits at 10–19 d; MRI 12–70 d; complete paraplegia; 100% mortality (sepsis/MOF)
Lindfors 2017 [24]	Simulation + case (Sweden)	Simulation + 1 clinical case	Drainage IVC vs RA vs SVC	At 4.2 L/min ECMO: predicted right-arm SaO_2_ 64% (IVC), 75% (RA), 81% (SVC); clinically SaO_2_ improved to >80–85% after RA then SVC drainage; discharged neurologically intact
Gan 2023 [21]	Prospective 2-case series (China)	2 adult women	Bifemoral VA	Serial Doppler mapping used to infer watershed migration with flow reduction and LV recovery
Rupprecht 2015 [26]	Retrospective cannulation analysis (Germany)	Percutaneous VA subset n = 159	Femoral VA; relocation to subclavian when needed	Harlequin/upper-body hypoxia 8.8% (~14/159) prompting subclavian relocation; limb ischemia 16.9% (7.5% surgical revision)
Salih 2024 [28]	Multicenter retrospective (Germany)	27 adults on VA-ECMO undergoing apnea testing	Femoro-femoral VA	PaCO_2_ difference at AT start: 0.9 mmHg (bilateral radials) vs 3.3 (radial vs post-oxygenator), *p* = 0.001; AT end: 1.1 vs 9.9 (max 26.7), *p* = 0.002; all AT completed without hemodynamic collapse

**Abbreviations**: ARDS, acute respiratory distress syndrome; AT, apnea testing; CEUS, contrast-enhanced ultrasound; CPC, Cerebral Performance Category; CT, computed tomography; CTA, computed tomography angiography; DH, differential hypoxemia; ECMO, extracorporeal membrane oxygenation; HTx, heart transplantation; IABP, intra-aortic balloon pump; ICU, intensive care unit; IJV, internal jugular vein; IVC, inferior vena cava; LV, left ventricular; LVAD, left ventricular assist device; LVEF, left ventricular ejection fraction; MAP, mean arterial pressure; MCS, mechanical circulatory support; MOF, multi-organ failure; MRI, magnetic resonance imaging; PaCO_2_, arterial partial pressure of carbon dioxide; PaO_2_, arterial partial pressure of oxygen; PAP, pulmonary artery pressure; RA, right atrium; SaO_2_, arterial oxygen saturation; SAVE, Survival After Venoarterial ECMO; SCI, spinal cord infarction; SpO_2_, peripheral oxygen saturation; SVC, superior vena cava; VA, venoarterial; VA-ECMO, venoarterial extracorporeal membrane oxygenation; V-AV, veno-arteriovenous; VV, venovenous. *Du et al. [19] was treated as a clinically adjacent retrograde-perfusion MCS analogue because it described watershed/mixing phenomena in retrograde-perfusion MCS configurations rather than providing direct peripheral VA-ECMO cohort evidence.

## 3. Results

### 3.1 Clinical Evidence: Incidence, Definitions, and Bedside Detection

Across clinical cohorts that reported denominators, the frequency of DH/Harlequin physiology varied by operational definition and population. In a 60-patient cardiac surgery/heart transplant cohort supported with femoro-femoral VA-ECMO, Harlequin syndrome occurred in 8/60 (13.3%) and prompted conversion strategies [18]. In a considerable percutaneous cannulation experience, “upper-body hypoxia/Harlequin syndrome” occurred in 8.8% and prompted escalation to subclavian arterial return relocation [26]. Overall, the management literature clustered into drainage optimization, circuit reconfiguration (V-AV with flow-split control), and proximal return relocation, with representative quantitative improvements summarized in Table 2 (Ref. [17,18,20,24,26])*.* In a high-volume VA-ECMO center cohort using a severe threshold definition (“fulminant DH”, including upper-body peripheral oxygen saturation (SpO_2_) ≤60% and/or clearly lower upper-body than lower-body oxygenation), fulminant DH occurred in 7/472 (1.5%) [20]. Note, however, that these values should not be interpreted as a pooled incidence estimate because they reflect different definitions, denominators, monitoring strategies, and clinical thresholds for recognition. Nevertheless, these results suggest that DH is commonly encountered when defined by clinically meaningful upper–lower oxygenation discordance, whereas extreme hypoxemic phenotypes are less frequent [18,20,26].

**Table 2.** **Differential hypoxemia mitigation strategies and quantitative effects**.

Strategy	Study	Key quantitative response signal(s)	Key clinical trajectory signal(s)
Drainage repositioning IVC→SVC/upper RA	Falk 2023 [20]	SpO_2_ 54 ± 6.6→86 ± 6.6; lactate 6.5→2.8 mmol/L at 24 h	5/7 survived; 2 deaths from intracerebral lesions
VA→V-AV with jugular return (double-pump control)	Contento 2020 [18]	PaO_2_ ratio 0.8 ± 0.1→0.95 ± 0.02; lactate 5.3→~0.9 mmol/L	7/8 staged VA→V-AV→VV then weaned; 1/8 returned to VA then weaned
V-AV with controlled flow diversion to prevent “steal”	Cakici 2018 [17]	Flow split ~70% IJV→34%; PaO_2_ ratio 0.51 ± 0.07→0.93 ± 0.02	8/12 weaned; 3 bridged; 4 deaths (sepsis/MOF)
Arterial return relocation to subclavian	Rupprecht 2015 [26]	Harlequin/upper-body hypoxia 8.8% (~14/159)	Return-site relocation used as escalation pathway
Drainage site manipulation (simulation + case)	Lindfors 2017 [24]	Predicted SaO_2_ 64% (IVC)→81% (SVC) at constant flow	Clinical SaO_2_ improved to >80–85%; discharged intact

**Abbreviations**: IJV, internal jugular vein; IVC, inferior vena cava; MOF, multi-organ failure; PaO_2_, arterial partial pressure of oxygen; RA, right atrium; SaO_2_, arterial oxygen saturation; SpO_2_, peripheral oxygen saturation; SVC, superior vena cava; VA, venoarterial; V-AV, veno-arteriovenous; VV, venovenous.

Detection most often began with recognition of upper-body versus lower-body oxygenation discordance using right upper-extremity (or right ear) pulse oximetry, followed by confirmatory arterial blood gas (ABG) measurements, consistent with the clinical framing of DH as a “dual-circulation”/mixing-interface problem in peripheral VA-ECMO [20,24]. Several studies moved beyond the “presence of discordance” to attempts to localize or infer the mixing interface. In a prospective feasibility/safety study of contrast-enhanced ultrasound (CEUS) in 10 bifemoral VA-ECMO patients, the aortic watershed was directly visualized in the abdominal aorta in 5/10 and inferred to be proximal/root (via continuous retrograde enhancement) in 5/10 [16]. A prospective two-case series described daily point-of-care ultrasound (POCUS) Doppler mapping (carotids, radials, abdominal aorta) to infer watershed migration in response to changes in LV pulsatility and ECMO flow adjustments [21]. A radiology case report highlighted a diagnostic pitfall in retrograde-perfusion MCS: a position-dependent “filling defect” appearance in the aortic root/ascending aorta on electrocardiogram (ECG)-gated computed tomography angiography (CTA) that resolved with repositioning and was interpreted as a watershed/mixing artifact rather than thrombus [19]. Together, these studies demonstrate a spectrum of bedside approaches, ranging from syndrome detection (upper–lower discordance) to watershed localization and the interpretation of imaging artifacts [16,19,21].

A clinically meaningful extension beyond oxygenation was demonstrated in adults undergoing brain death determination on VA-ECMO, in which Harlequin physiology was identified as a potential source of discordance in PaCO_2_ exposure during apnea testing. In 27 adults undergoing apnea testing, PaCO_2_ differences were minor when comparing simultaneous bilateral radial samples but substantially larger when comparing right radial PaCO_2_ to post-oxygenator PaCO_2_, with differences widening by the end of the apnea test [28].

### 3.2 DH Management Strategies

The pathophysiologic basis of differential hypoxemia and the principal configuration-based strategies used to restore upper-body oxygen delivery are summarized schematically in Fig. 2. In the fulminant DH cohort, adding or repositioning drainage toward the superior vena cava (SVC)/upper right atrium via a right internal jugular drainage cannula was associated with marked improvement in upper-body oxygenation (upper-body SpO_2_ rising from 54 ± 6.6% to 86 ± 6.6%, *p* < 0.001), improved pre-oxygenator saturation (62 ± 8.9% to 74 ± 3.7%, *p* = 0.016), and less negative pre-pump pressures (−66 ± 35 to −31 ± 37 mmHg, *p* = 0.037) [20]. Convergent mechanistic support came from simulation + case evidence demonstrating that superior vena cava drainage increased right-arm saturation without changing ECMO flow, consistent with a venous “source” contribution to the oxygen content of blood ejected by the LV into the arch [24].

**Fig. 2.**
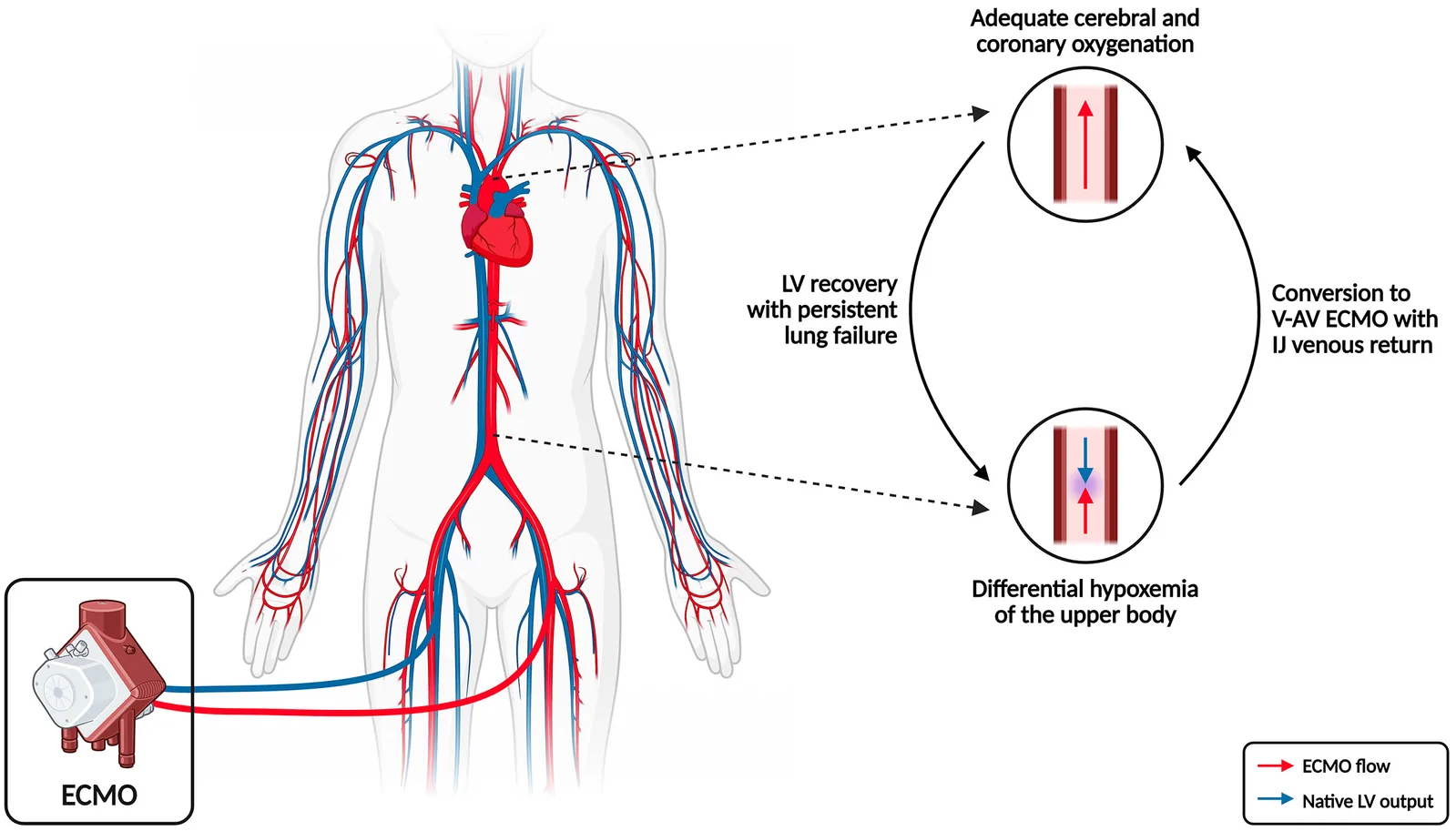
**Simplified schematic of the aortic watershed phenomenon and differential hypoxemia (Harlequin/north-south syndrome) during peripheral femoro-femoral VA-ECMO**. In peripheral VA-ECMO, oxygenated blood returned retrogradely via the femoral arterial cannula competes with antegrade native left ventricular (LV) ejection, creating a dynamic aortic mixing interface (“watershed”). When lung failure persists while LV ejection recovers, the LV may eject poorly oxygenated blood into the proximal aorta, shifting the watershed distally and predisposing the coronary and supra-aortic circulations (brain) to preferential perfusion by native (hypoxemic) blood, manifesting clinically as upper-body hypoxia despite preserved distal (lower-body) oxygenation from ECMO flow. Mechanism-based strategies to restore upper-body oxygen delivery include adding internal jugular (IJ) venous drainage to shift drainage toward the SVC/upper right atrium (SVC/RA) and/or converting to veno-arteriovenous (V-AV) ECMO with IJ return. Figure created with BioRender.

In a cohort where Harlequin syndrome triggered conversion to a double-pump V-AV configuration with internal jugular return, physiologic indices improved and upper–lower oxygenation ratios normalized: upper/lower arterial partial pressure of oxygen (PaO_2_) ratio improved (0.8 ± 0.1 to 0.95 ± 0.02) and upper/lower arterial oxygen saturation (SaO_2_) ratio normalized (0.83 ± 0.02 to 1.0), with concurrent improvements in hemodynamics and lactate [18]. Furthermore, in a 12-patient V-AV series describing outflow steal, actively controlling the return split shifted flow from the internal jugular limb to the femoral arterial limb, coinciding with improved upper- and lower-body oxygenation ratios and metabolic markers [17]. In a large percutaneous cannulation series, Harlequin/upper-body hypoxia was documented as an escalation-triggering event (8.8%), prompting relocation of arterial return to the subclavian artery [26].

### 3.3 Clinical Outcomes and Complications

Neurologic complications were reported in association with severe DH and peripheral VA-ECMO watershed physiology. In the fulminant DH cohort, two patients died with intracerebral lesions despite improved oxygenation after drainage repositioning [20]. Separately, a case series reported spinal cord infarction in 3 of 504 peripheral VA-ECMO runs (0.6%), with delayed recognition and death from multiorgan failure/sepsis in all three cases [22]. These observational data support clinical concern but do not establish DH as the sole cause of neurologic injury [20,22].

### 3.4 Experimental Evidence

Experimental studies clarified why DH can become refractory to ECMO flow escalation when native LV output increases, and pulmonary gas exchange remains poor. Key mechanistic findings from computational and *in vitro* models are summarized in Table 3 (Ref. [23,25,27,29,30,31]). Patient-specific modelling demonstrated that as native LV stroke volume increases, the mixing interface can shift such that arch vessels become increasingly perfused by native output rather than ECMO-origin blood, limiting the ability of “more ECMO flow” alone to correct arch oxygenation [29]. Complementary oxygen-transport computational fluid dynamics (CFD) modelling showed that increasing native cardiac output under VA-ECMO support can reduce aortic/arch oxygen distribution when pulmonary oxygenation is absent or severely impaired, consistent with DH worsening during cardiac recovery if lungs remain failing [30].

**Table 3.** **Experimental studies: key mechanistic findings relevant to clinical differential hypoxemia**.

Study	Model	Key variation	Key quantitative finding(s)
Stevens 2018 [29]	Patient-specific CFD framework	LV stroke volume vs ECMO flow	At total flow 5 L/min, BCA ECMO fraction ~70%→0% as SV 4→16 mL; no ECMO to arch when SV >28 mL
Zhang 2018 [30]	CFD + oxygen transport	LV fraction 0→50% of total	Brachiocephalic SaO_2_ fell to ~73.6–70.5% at LV 2–2.5 L/min (of 5 L/min total)
Malinowski 2023 [25]	CFD with coronaries	Cannula tip position	Ascending-tip: RCA ~97% and ≥90% arch branches at partial support; descending-tip could leave RCA low despite supra-aortic 92–97%
Feiger 2024 [31]	Whole-body computational modeling	Anatomy + ECMO support fraction	VA-ECMO ≥50% in MCA required ~67–97% support; RMCA threshold higher (~80–96%); total CBF decreased ~5.8–20.6% with increasing support
Gehron 2020 [23]	Mock circulation loop	Cannulation site and LV:ECLS ratio	Femoral return produced thoracic mixing cloud with near-stagnant velocities (0 ± 4 and −10 ± 15 cm/s) and limited migration of isthmus watershed
Rozencwajg 2024 [27]	Mock circulation loop	MZ location and detection signal	Pulse pressure failed to localize MZ (r^2^ ≈ 0); temperature/thermodilution signals distinguished brain ECMO-perfused vs LV-perfused states

**Abbreviations**: BCA, brachiocephalic artery; CBF, cerebral blood flow; CFD, computational fluid dynamics; ECLS, extracorporeal life support; LV, left ventricular; MCA, middle cerebral artery; MZ, mixing zone; RCA, right coronary artery; RMCA, right middle cerebral artery; SaO_2_, arterial oxygen saturation; SV, stroke volume; VA-ECMO, venoarterial extracorporeal membrane oxygenation.

Whole-body (1D) modelling across multiple geometries suggested that the ECMO support fraction required to perfuse the cerebral circulation with ECMO-origin blood adequately is anatomy-dependent and can be very high; across eight semi-idealized geometries, the VA-ECMO support fraction required to meet predefined cerebral perfusion thresholds varied widely and consistently required higher support for the right middle cerebral artery than the left [31]. CFD studies incorporating endoluminal arterial cannulation geometry suggested coronary oxygenation may not track supra-aortic branch oxygenation under some configurations; cannula placement more proximal in the aorta improved oxygenation of multiple aortic branches compared with more distal placements, with implications for myocardial risk when the mixing interface lies distal to the coronaries [25].

*In vitro* mock-circulation loop studies provided direct visualization and measurement of mixing behaviour. A systematic *in vitro* investigation characterized watershed phenomena and flow distribution patterns during extracorporeal life support, supporting the concept that mixing can involve broader regions rather than a thin plane, and that cannulation routes and native/ECMO flow interactions materially influence flow phenomena [23]. A separate mock circulation loop study validated an *in vitro* DH model and found that pulse pressure did not reliably localize the mixing zone. In contrast, thermodilution-based approaches could distinguish whether cerebral perfusion was dominated by ECMO-origin flow [27].

## 4. Discussion

Based on clinical and experimental evidence, the watershed phenomenon in peripheral femoro-femoral VA-ECMO can be understood as a competitive interaction between retrograde ECMO return and antegrade native LV ejection, creating a mixing interface whose position determines whether the supra-aortic branches and coronaries receive predominantly ECMO- or native LV-origin blood [24,29,30]. DH develops when pulmonary gas exchange remains poor. At the same time, LV ejection persists or recovers, allowing relatively desaturated (and potentially CO_2_-rich) native blood to reach the ascending aorta and arch territories despite adequate lower-body perfusion from femoral ECMO return [20,24,30].

A central mechanistic implication from modelling is that DH may become “flow-refractory” as native LV contribution rises. Patient-specific CFD work demonstrated that increasing LV stroke volume can markedly reduce the fraction of ECMO-origin flow reaching arch branches, implying that escalating ECMO flow alone may not reliably restore cerebral/coronary oxygenation when pulmonary failure persists [29]. Oxygen-transport CFD similarly demonstrated reduced aortic oxygen distribution with increasing native cardiac output under VA-ECMO when lung oxygenation is absent/severely impaired, consistent with clinical observation that DH may emerge during cardiac recovery [30].

Attempts to correct DH by simply increasing VA-ECMO flow may be physiologically limited when native left ventricular output is recovering, but pulmonary gas exchange remains impaired, because deoxygenated native blood may continue to supply the proximal aorta and arch vessels despite extracorporeal support [29,30]. Although changes in ECMO flow can alter the position of the mixing interface, isolated flow escalation does not directly correct the pulmonary substrate that determines the oxygen content of native left ventricular output [29,30]. In addition, higher VA-ECMO support can increase left ventricular afterload, promote left ventricular distension, and transmit elevated left-sided filling pressures to the pulmonary circulation, thereby contributing to pulmonary congestion and potentially worsening the respiratory substrate for upper-body hypoxemia [32]. Persistent or severe DH should therefore prompt mechanism-based reassessment rather than reflexive escalation of ECMO flow alone. If the dominant issue is inadequate capture of desaturated upper-body venous return, shifting drainage toward the superior vena cava/upper right atrium is supported by clinical and simulation evidence [20,24]. If severe pulmonary dysfunction persists despite adequate circulatory support, conversion to veno-arteriovenous ECMO may improve upper–lower oxygenation balance, particularly when flow partitioning is actively monitored and controlled to avoid preferential venous return “steal” [17,18]. When upper-body hypoxemia remains a concern in femoro-femoral VA-ECMO, arterial return relocation to a more proximal or antegrade site may be considered as an escalation strategy in selected patients [26].

Furthermore, anatomical dependence likely contributes to inter-patient variability. Whole-body (1D) modelling across multiple geometries showed that achieving adequate cerebral perfusion with ECMO-origin flow may require a high support fraction. Right-sided cerebral perfusion can be more difficult to “capture” with retrograde femoral return, supporting a physiologic rationale for right-arm/right-radial monitoring as a surrogate for vulnerable supra-aortic exposure [31]. Lastly, coronary vulnerability is mechanistically plausible when the mixing interface lies distal to the coronary ostia. CFD analyses of endoluminal arterial perfusion suggest that proximal cannula placement strategies can improve oxygenation of key proximal branches compared with distal placement, reinforcing the concern that “acceptable limb saturation” does not necessarily guarantee coronary protection across all geometries [25].

Clinically, DH most often presents as a marked oxygen saturation gradient between right upper-body sites (right hand/right ear/right radial ABG) and lower-extremity sites in femoro-femoral VA-ECMO, reflecting differential exposure of arch territories versus distal aorta territories to native versus ECMO-origin blood [20,24,26]. Frequency estimates varied by definition and denominator: 13.3% in a Harlequin-defined cohort [18], 8.8% as an escalation-triggering “upper-body hypoxia/Harlequin” event in a cannulation series [26], and 1.5% for a severe “fulminant DH” phenotype [20]. Importantly, the syndrome is not limited to oxygenation. Brain-death determination data demonstrate that PaCO_2_ exposure can vary meaningfully across sampling sites during VA-ECMO; a systematic study showed greater PaCO_2_ discordance when comparing arterial samples to post-oxygenator samples than when comparing bilateral radial arterial samples, implying that sampling strategy materially affects the interpretation of apnea testing in the presence of mixing physiology [28].

Routine monitoring during peripheral femoro-femoral VA-ECMO should prioritize the vascular territory most likely to reflect proximal aortic and supra-aortic oxygenation, particularly the right radial arterial line and right hand or right upper-torso pulse oximetry [2]. Femoral, lower-extremity, and post-oxygenator measurements may confirm distal or circuit oxygenation but do not reliably exclude cerebral or coronary hypoxemia when dual-circulation physiology is present [6,28].

The same sampling principle applies to PaCO_2_ interpretation during apnea testing, where post-oxygenator or distal arterial values may not reflect gas tensions in the cerebral circulation when arterial mixing is incomplete [28]. Cerebral and peripheral near-infrared spectroscopy may provide useful continuous adjunctive information on brain and limb perfusion, but should be interpreted alongside arterial blood gases, pulse oximetry, cannulation configuration, and native cardiac output [2]. Continuous electrocardiographic monitoring is also appropriate, as proximal aortic hypoxemia may affect the coronary circulation [2,6].

Across clinical reports, severe lung dysfunction with preserved or recovering LV ejection was the recurring substrate for DH, consistent with the mechanism that native blood ejected into the arch remains poorly oxygenated. At the same time, ECMO perfuses the distal aorta retrogradely [20,24,29]. The venous drainage position appears to be contributory, as both cohort and simulation/case evidence support the concept that inferior vena cava (IVC)-dominant drainage may fail to capture the most desaturated SVC blood. This allows severely desaturated SVC-derived venous return to pass through the right heart, lungs, and LV, thereby sustaining hypoxemic arch perfusion [20,24].

Prolonged proximal aortic hypoxemia may expose the brain and myocardium to desaturated native blood when the mixing interface lies distal to the supra-aortic branches or coronary ostia [2,6,25,31]. Neurologic injury has been reported in association with severe DH and peripheral VA-ECMO watershed physiology, including intracerebral lesions in fulminant DH and rare cases of spinal cord infarction during femoro-femoral VA-ECMO [20,22]. These observations should be interpreted cautiously because neurologic outcomes during VA-ECMO are strongly confounded by the severity of illness, cardiac arrest, embolic events, anticoagulation, duration of support, and systemic hypoperfusion [15,22]. Accordingly, the present review frames neurologic and myocardial injury as biologically plausible consequences of prolonged proximal hypoxemic exposure rather than outcomes definitively caused by DH alone [15,22].

Across included management studies, the most consistently supported interventions were those that modify venous sampling and/or circuit geometry to change the oxygen content and distribution of blood reaching the LV and arch territories, rather than relying exclusively on increasing ECMO flow [20,24,29,30]. In fulminant DH, shifting drainage toward the SVC/upper right atrium (RA) produced a significant and rapid upper-body oxygenation improvement with favourable short- and longer-term survival in most cases. However, a severe neurologic injury still occurred in some [20]. Simulation and case evidence support the mechanism whereby SVC drainage increases upper-body saturation without altering flow, consistent with the capture of desaturated venous return that would otherwise reach the LV and arch [24].

Persistent right radial hypoxemia in the setting of severe pulmonary dysfunction and recovering native left ventricular ejection should prompt early reassessment of whether conventional femoro-femoral VA-ECMO remains the optimal configuration [2,18]. In selected patients, earlier conversion to V-AV ECMO may be preferable to repeated escalation of VA-ECMO flow alone because it can provide oxygenated venous return to the upper circulation while maintaining arterial circulatory support [2,18]. When V-AV ECMO is used, flow partitioning should be actively monitored and adjusted, as preferential diversion to the venous return limb can reduce effective arterial support [17]. Furthermore, return-site relocation to a more proximal/antegrade arterial site (e.g., subclavian) is documented as a real-world escalation pathway when upper-body hypoxia/Harlequin physiology occurs [26].

Beyond conventional upper- and lower-body oximetry/ABG-based recognition, CEUS and POCUS Doppler mapping represent promising bedside localization approaches. CEUS directly visualized or inferred watershed position in bifemoral VA-ECMO patients in a feasibility study [16], and Doppler mapping demonstrated serial monitoring of watershed migration in a prospective POCUS series [21]. *In vitro* work suggests that hemodynamic surrogates, such as pulse pressure, may not reliably localize the mixing zone. In contrast, thermodilution-based signals may provide useful discriminative information about the origin of cerebral perfusion [27].

### Limitations

This scoping review is limited by heterogeneous definitions of DH, variable detection strategies, and predominantly observational clinical evidence, which constrain causal inference regarding the DH-specific contribution to neurologic outcomes [15]. Future work should prioritize: (1) standardized DH definitions that incorporate both oxygenation and CO_2_ discordance; (2) prospective validation of bedside localization tools (e.g., CEUS, Doppler mapping) against clinically meaningful endpoints; and (3) comparative effectiveness studies of drainage repositioning, V-AV conversion with flow control, and arterial return relocation using harmonized neurologic and myocardial outcome measures [16,17,18,20,28,29]. Furthermore, no formal critical appraisal or risk-of-bias assessment was performed; therefore, the synthesis should be interpreted as an evidence map rather than a graded certainty assessment. Accordingly, quantitative estimates, management responses, and neurologic outcome signals should be considered hypothesis-generating and should not be interpreted as pooled incidence estimates or causal effect estimates [15].

## 5. Conclusion

The aortic watershed phenomenon during peripheral femoro-femoral VA-ECMO reflects an unstable balance between retrograde circuit flow and native left ventricular ejection. When pulmonary gas exchange remains poor, recovery of native cardiac output can shift hypoxic blood toward the proximal aorta, placing cerebral and myocardial oxygen delivery at risk despite preserved lower-body oxygenation. The current evidence base remains limited by heterogeneous definitions, small clinical series, and experimental models, yet it consistently points toward physiology-guided management rather than simple escalation of ECMO flow. Careful monitoring of the right upper body, reassessment of venous drainage and arterial return, and timely circuit modification may improve recognition and treatment. Prospective studies are needed to standardize definitions, validate bedside localization tools, and evaluate clinically meaningful outcomes.
